# Nitration of Wheat Amylase Trypsin Inhibitors Increases Their Innate and Adaptive Immunostimulatory Potential *in vitro*

**DOI:** 10.3389/fimmu.2018.03174

**Published:** 2019-01-21

**Authors:** Kira Ziegler, Jan Neumann, Fobang Liu, Janine Fröhlich-Nowoisky, Christoph Cremer, Joachim Saloga, Kathrin Reinmuth-Selzle, Ulrich Pöschl, Detlef Schuppan, Iris Bellinghausen, Kurt Lucas

**Affiliations:** ^1^Multiphase Chemistry Department, Max Planck Institute for Chemistry, Mainz, Germany; ^2^Institute of Molecular Biology, Mainz, Germany; ^3^Department of Dermatology, University Medical Center of the Johannes Gutenberg University, Mainz, Germany; ^4^Institute of Translational Immunology, University Medical Center of the Johannes Gutenberg University, Mainz, Germany

**Keywords:** allergy, amylase trypsin inhibitors, dendritic cells, non-celiac wheat sensitivity, protein nitration, wheat

## Abstract

Amylase trypsin inhibitors (ATI) can be found in all gluten containing cereals and are, therefore, ingredient of basic foods like bread or pasta. In the gut ATI can mediate innate immunity via activation of the Toll-like receptor 4 (TLR4) on immune cells residing in the lamina propria, promoting intestinal, as well as extra-intestinal, inflammation. Inflammatory conditions can induce formation of peroxynitrite (ONOO^−^) and, thereby, endogenous protein nitration in the body. Moreover, air pollutants like ozone (O_3_) and nitrogen dioxide (NO_2_) can cause exogenous protein nitration in the environment. Both reaction pathways may lead to the nitration of ATI. To investigate if and how nitration modulates the immunostimulatory properties of ATI, they were chemically modified by three different methods simulating endogenous and exogenous protein nitration and tested *in vitro*. Here we show that ATI nitration was achieved by all three methods and lead to increased immune reactions. We found that ATI nitrated by tetranitromethane (TNM) or ONOO^−^ lead to a significantly enhanced TLR4 activation. Furthermore, in human primary immune cells, TNM nitrated ATI induced a significantly higher T cell proliferation and release of Th1 and Th2 cytokines compared to unmodified ATI. Our findings implicate a causative chain between nitration, enhanced TLR4 stimulation, and adaptive immune responses, providing major implications for public health, as nitrated ATI may strongly promote inhalative wheat allergies (baker's asthma), non-celiac wheat sensitivity (NCWS), other allergies, and autoimmune diseases. This underlines the importance of future work analyzing the relationship between endo- and exogenous protein nitration, and the rise in incidence of ATI-related and other food hypersensitivities.

## Introduction

Nitration of proteins can occur endogenously in the human body or exogenously in the environment. Air pollutants, like ozone and nitrogen oxides, are able to nitrate allergenic proteins, like the major birch pollen allergen Bet v 1, leading to an increased allergic potential and elevated immune reactions (1–6). In the human body, inflammatory conditions can lead to the formation of ONOO^−^, which is the main agent for endogenous protein nitration (5, 7–9). Moreover, several air pollutants and pathogens can induce or favor inflammatory processes and related nitration processes (1, 3, 6). Diseases typically accompanied by a high degree of inflammation are, for example, autoimmune and other chronic inflammatory diseases, like chronic obstructive pulmonary disease (COPD), allergic asthma, inflammatory bowel diseases (IBD), or non-celiac non-allergy wheat sensitivity (NCWS) (10–12).

We previously showed that amylase trypsin inhibitors (ATI), a family of non-gluten proteins, are responsible for manifestations of mainly extra-intestinal symptoms of NCWS (10, 11, 13, 14). Moreover, ATI are major allergens in baker's asthma, a classical IgE mediated allergy (15, 16). ATI can be found in all gluten containing grains (wheat, barley, rye), and represent 2–4% of the total protein (17). In plants, ATI regulate the germination processes (18) and defense mechanisms by blocking the amylase and trypsin activity of parasites (19). The wheat intake of an adult person is about 250 g per day, mainly as processed bread or pasta which is equivalent to 0.5–1 g ATI (10). Remarkably, ATI are resistant to food processing and to proteolysis in the gastrointestinal tract where they stay biologically active (20). In the gut ATI are able to stimulate immune cells residing in the lamina propria and mesenteric lymph nodes through TLR4 binding and stimulation, and the emigration of the activated myeloid cells (20–22).

The innate immune receptor TLR4 recognizes damage and pathogen associated patterns (DAMPs/PAMPs), like lipopolysaccharides (LPS), which are major components in the outer membrane of gram negative bacteria. Upon stimulation, the receptor triggers an NF-κB dependent cascade leading to the release of pro-inflammatory cytokines (23, 24). Importantly, ATI can trigger TLR4 by direct interaction, and provoke innate immunity (21, 22). In a mouse model of inflammatory bowel disease, ATI enhanced the dextran sodium sulfate-induced intestinal inflammation by increasing the number of activated macrophages and dendritic cells in all sections of the intestine, the lamina propria, and especially in the mesenteric lymph nodes (20). Moreover, we showed in two mouse studies on experimental airway inflammation that ATI-enriched diets not only enhanced allergen-induced intestinal, but also lung allergic responses in an IgE- and TLR4-dependent manner (25, 26). Thus, the adjuvant effect of ATI is not limited to the intestine, but can also be observed for other organs, fueling ongoing inflammation. These inflammatory conditions might favor the formation of ONOO^−^ and, thereby, protein nitration (7–9, 27), including ATI in the gut, apart from the environmental factors that may induce protein nitration.

To elucidate the effect of nitration, we chemically modified ATI using three different methods simulating endo- (*in vivo*) and exogenous (environmental) nitration mechanisms. These nitrating agents mainly induce the formation of 3-nitrotyrosine (1, 5, 27–31). The aim of our study was to examine whether nitrated ATI exhibit an altered innate and adaptive immunostimulatory capacity. Modified ATI were quantified for their nitration degree using HPLC-DAD and ELISA. Furthermore, various *in vitro* studies were performed. A novel HeLa TLR4 reporter cell line was established for determination of innate immune stimulation. Adaptive immune reactions were analyzed in a mixed lymphocyte reaction by the use of primary immune cells isolated from whole blood of healthy donors. TLR4 activation, NF-κB translocation, expression of surface maturation markers, Th1, Th2, relevant cytokines, and T cell proliferation were measured.

## Materials and Methods

### Nitration and Analysis of Nitrated ATI

#### Nitration With Tetranitromethane (TNM) of ATI

ATI were obtained from Sigma Aldrich (α-Amylase Inhibitor from *Triticum aestivum* (wheat seed), Sigma-Aldrich, Darmstadt, Germany). Aliquots of aqueous ATI solutions (1 mg/mL, 0.5 mL) were mixed with 4.55 μL TNM/Methanol (4%, v/v) and stirred for 3 h at room temperature. To remove excess TNM after the reaction, a PD-25 size exclusion chromatography column (GE Healthcare, Little Chalfont, Great Britain) was used according to the manufacturer's instructions. Finally, the ATI were eluted with endotoxin free water (MilliQ, Biopak, Merck, Darmstadt, Germany).

#### Nitration With O_3_/NO_2_ of ATI

ATI aqueous solutions (0.5 mg/mL, 1 mL) were exposed to a gas mixture of O_3_ and NO_2_, as described previously (31). Briefly, O_3_ was produced from synthetic air passed through a UV lamp (LOT-Quantum Design, Darmstadt, Germany) at ~1.98 L/min. The air flow was then mixed with a N_2_ flow (20 mL/min) containing ~5 ppmV NO_2_ (AirLiquide, Düsseldorf, Germany). The resulting air gas mixtures were bubbled directly through the aqueous ATI solutions at a flow rate of 60 mL/min using a Teflon tube (ID: 1.59 mm). The concentrations of O_3_/NO_2_ were monitored by commercial monitoring instruments (Ozone analyzer, 49i; NO_x_ analyzer, 42i-TL, Thermo Fisher Scientific, Darmstadt, Germany, respectively).

#### ONOO^−^ Nitration of ATI

ATI solutions were prepared in 50 mM ammonium bicarbonate buffer at pH 7.8 (Carl Roth, Karlsruhe, Germany). For one reaction 300 μL of ATI solution [1 mg/mL] were mixed with 2.85 μL ONOO^−^ (160–200 mM, Merck) in brown reaction vessels (Eppendorf, Hamburg, Germany) and incubated for 110 min on ice. Immediately after the reaction, the samples were desalted using a PD-10 size exclusion mini column (GE Healthcare), following the manufacturer manual with endotoxin free water.

#### HPLC-DAD Analysis

All nitrated ATI samples were analyzed using HPLC coupled to diode array detection (HPLC-DAD, 1,200 series, Agilent Technologies, Santa Clara, California, USA). Values were determined using peak areas of signals at wavelengths 280 and 357 nm. A detailed description of the analytical method can be found in Selzle et al. (30). The nitration degree is defined as the concentration of nitrotyrosine as a fraction of the sum of the concentrations of nitrotyrosine and tyrosine. For example, ATI 0.19 Chain D from *T. aestivum* comprises five tyrosine residues. A nitration degree of 20% reflects on average one nitrotyrosine per ATI 0.19 molecule.

#### Endotoxin Quantification

Endotoxin was quantified by Limulus Amebocyte Lysate chromogenic endotoxin quantitation kit (Thermo Fisher Scientific). ATI samples were tested at several dilutions and compared to an *Escherichia coli* endotoxin standard (011:B4) provided with the kit. The endotoxin levels in the final concentration used for all experiments were < 20 Endotoxin units per mL.

#### Protein Analysis

To determine the ATI protein concentrations before and after nitration, a bicinchoninic acid assay (Thermo Fisher Scientific) was used according to the manufacturer's instructions. The optical density at 562 nm was determined using a Synergy Neo plate reader (Biotek, Bad Friedrichshall, Germany).

ATI oligomers were detected using sodium dodecyl sulfate polyacrylamide gel electrophoresis (5–20%, Bio-Rad, Munich, Germany), referring to the instruction manual. A 5 μg portion of each sample was prepared in 2x Laemmli buffer (Bio-Rad) containing 100 mM Dithiothreitol (Sigma Aldrich), heated on 96°C for 5 min, and loaded on the gel. After separation, the gel was stained for 3 h in Coomassie blue (Bio-Rad) and unstained in an aqueous solution containing 10% methanol (Merck) and 20% acetic acid (Carl Roth) over night. For image acquisition and for analysis, a ChemiDoc system and Image Lab software 5.2.1 (both Bio-Rad) were used, respectively.

### Cell Culture

#### Hela TLR4 Dual Luciferase Reporter Cell Line (HeLa TLR4 Dual)

Cells were grown in Dulbecco's Modified Eagle's Medium (DMEM, Thermo Fisher Scientific) containing 25 mM D-glucose, 1 mM sodium pyruvate supplemented with 10% heat-inactivated fetal calf serum (FCS), (Biochrom, Berlin, Germany), 1% Penicillin/Streptomycin (Thermo Fisher Scientific), and 140 μg/mL Hygromycin B (Invivogen, Toulouse, France) in a humidified atmosphere of 5% CO_2_ at 37°C.

For simultaneous determination of TLR4 stimulation and viability, a novel monoclonal dual reporter cell line was established. Therefore, the HeLa TLR4 cell line (Novusbio, Wiesbaden, Germany), expressing Renilla luciferase under the control of an IL-8 promotor reporting TLR4 activity, was stably transfected with a plasmid, constitutively expressing Firefly luciferase, and, thereby, measuring viability (pCMB-firefly-luc-hygro, kindly provided by Ernesto Bockamp, University Medical Center of the Johannes Gutenberg University). Lipofectamine 3000 (Thermo Fisher Scientific) was used as the transfecting reagent according to the manufacturer's protocol.

##### Combined TLR4 and viability assay

20,000 HeLa TLR4 dual reporter cells were seeded in a flat bottom 96-well plate (Greiner, Frickenhausen, Germany) in 100 μL complete DMEM. On the next day, the cells were treated with different nitrated ATI at a final concentration of 7.5 μg/mL. Mock nitrations and medium served as negative controls, and LPS EB (25 ng/mL, Invivogen) as a positive control. After 7 h, the plate was washed with 200 μL of warm PBS containing calcium and magnesium (Thermo Fisher Scientific). Then the cells were lysed by adding passive lysis buffer (Dual-luciferase reporter assay, Promega, Mannheim, Germany) and frozen at −80°C. An analysis of both luciferase reporter activities in the cell lysate was performed according to the manufacturer's manual (Promega). The relative luciferase activity was calculated by dividing the Renilla luciferase (TLR4) signal by the Firefly luciferase (viability) signal. The resulting values were normalized to the value obtained for LPS treated cells.

To inhibit TLR4 signaling, HeLa TLR4 dual cells were pre-incubated with the TLR4 antagonist TAK242 (0.36 μg/mL, Merck), or, as a negative control, its solvent dimethylsulfoxide (4.4 μg/mL, Thermo Fisher Scientific) for 150 min.To provide a stronger stimulation, the doses of ATI were doubled in these experiments [15 μg/mL].

#### Generation of Monocyte Derived Dendritic Cells (DC)

Buffy coats from ten healthy donors were obtained from the Transfusion Center (University Medical Center of the Johannes Gutenberg University, Mainz, Germany) with approval from the local ethical committee (Landesärztekammer Rheinland-Pfalz). Peripheral blood mononuclear cells (PBMC) were isolated by Ficoll-Paque 1.077 g/mL (Biochrom) density centrifugation. The autologous plasma was heat-inactivated at 56°C for 30 min, centrifuged at 1,500 × g, and stored at 4°C. To enrich CD14^+^ monocytes, 5 × 10^6^ PBMC per well were incubated in a 12-well plate (Greiner) in 1.5 mL Iscove modified Dulbecco medium (IMDM, Lonza, Basel, Switzerland) enriched with 1% antibiotic/antimycotic solution (Sigma Aldrich) and 3% autologous plasma for 40 min in a cell incubator under 5% CO_2_ atmosphere at 37°C. Cells were washed 3 times with warm PBS (without calcium magnesium, Thermo Fisher Scientific), and maintained in IMDM, supplemented with 10 ng/mL IL-4 (Miltenyi, Bergisch Gladbach, Germany), 200 U/mL granulocyte-macrophage colony-stimulating factor (GM-CSF, Leukine®, Immunex Corp., Seattle, WA, USA), and 2% autologous plasma. On day 6, immature DC were pulsed with ATI [15 μg/mL], TNM nitrated ATI [15 μg/mL], equivalent amounts of mock nitrated solution, or were left untreated. To induce DC maturation, the cells were additionally treated with tumor necrosis factor (TNF)-alpha, 10 ng/mL, Miltenyi Biotec), IL-1β (10 ng/mL, Miltenyi Biotec) and prostaglandin E_2_ (1 μg/mL, Cayman Chemical, Ann Arbor, MI, USA). After 48 h, DC were harvested, washed twice in cold PBS, and used for T cell stimulation assays as well as for analysis of surface marker expression.

##### Surface marker staining and analysis by flow cytometry

5 × 10^4^ DC or 5 × 10^5^ T cells were stained with specific mouse anti-human monoclonal antibodies (mAbs) for 20 min at 4°C. The following antibodies were used: AlexaFluor 647-conjugated CD4 (MT310; Santa Cruz Biotechnology, Inc., Santa Cruz, CA, USA), fluorescein isothiocyanate (FITC)-conjugated human leukocyte antigen D-related (HLA-DR) (L243), phycoerythrin (PE)-conjugated CD80 (L307.4), and allophycocyanin-conjugated CD83 (HB15e, all from BD Biosciences). As a negative control, matured cells were used. Then, cells were washed, and analyzed by BD Accuri™ C6 Plus Flow Cytometer (BD Biosciences).

##### Isolation of CD4^+^ T cells and co-culture with autologous native ATI- or TNM nitrated ATI-pulsed DC

Autologous CD4^+^ T cells were obtained from PBMC using antibody-coated paramagnetic MicroBeads (MACS; Miltenyi Biotec) according to the manufacturer's protocol. Separation was confirmed by flow cytometry (purity, >98% CD4^+^ T cells).

1 × 10^5^ T cells and 1 × 10^4^ mDC were co-cultured in 96-well plates (Greiner) in triplicates in 200 μL IMDM containing 5% autologous plasma. Five days later, 50 μL of supernatant was taken for quantification of cytokine production. For the determination of proliferation, the co-culture was pulsed with 37 kBq/well of [^3^H]-thymidine (ICN Biomedicals, CA, USA) for 6 h and [^3^H]-TdR incorporation was measured using a beta counter (1205 Betaplate, LKB Wallac, Turku, Finland).

##### Quantification of soluble cytokines by magnetic multiplex assay

For quantification of cytokines, multiplex assay kits (R&D systems, Biotechne, Wiesbaden, Germany) were used. Supernatants from immature DC cultures were tested for TNF-alpha, IL-6, IL-8, IL-1β, and monocyte chemoattractant protein 1 (MCP1). Supernatants of mature DC-T cell co-cultures were analyzed for interferon gamma (IFN-gamma), IL-10, IL-17, IL-6, IL-4, IL-5, and IL-13. The samples were prepared according to the manufacturer's manual and analyzed on a MAGPIX device (Luminex, Austin, Texas, USA).

### Quantification of ATI Induced NF-κB Translocation

Nuclear NF-κB translocation in macrophages was determined by fluorescence microscopy. Therefore, PBMCs were isolated and seeded in 12-well glass bottom plates (Cellvis, California, USA) at a concentration of 1.5 × 10^6^ – 2 × 10^6^ cells per well in IMDM enriched with 200 U/mL GM-CSF and 2% autologous plasma. After 6 days, cells were stimulated for 2 h either with ATI, ATI TNM [12.5 μg/mL], or with LPS EB (100 ng/mL, Invivogen) as a positive control. Untreated cells (medium) served as a negative control. Next, cells were washed once in pre-warmed PBS and fixed using 4% formaldehyde solution (Thermo Fisher Scientific) in PBS for 10 min at 37°C. Subsequently, cells were rinsed three times with PBS, then blocked and permeabilized in PBS containing 0.3% Triton X-100 (Merck) and 5% bovine serum albumin (BSA, Cell Signaling Technology, Danvers, Massachusetts) for 1 h at room temperature. Afterwards, cells were incubated with primary rabbit anti-NF-κB p65 mAb (D14E12, Cell Signaling Technology) diluted in PBS comprising 1% BSA and 0.3% Triton X-100 (Merck) overnight at 4°C. Thereafter, cells were washed three times in PBS for 5 min each and incubated for 1 h at room temperature with anti-rabbit Alexa Fluor 568 antibody (A-11011, Thermo Fisher Scientific) diluted in PBS comprising 1% BSA and 0.3% Triton X-100. Cells were washed again three times in PBS for 5 min each and cell nuclei were counterstained using 4′,6-diamidino-2-phenylindole (DAPI, Thermo Fisher Scientific) according to the manufacturer's instructions. Imaging of stained macrophages was performed at an Opera Phenix High-Content Screening system (Perkin Elmer, Waltham, Massachusetts, USA). PBS was used as an imaging buffer. For image analysis, Harmony high-content analysis software (Perkin Elmer, Waltham, Massachusetts, USA) was used. Nuclei were identified by DAPI staining, and a ring-like mask around each identified nucleus was created to obtain the corresponding cytoplasm area. Afterwards, the mean intensities of NF-κB within both masks were determined and the nuclear to cytoplasmic ratio of NF-κB was calculated. The described image processing steps are illustrated in Figure S1.

### Statistics

GraphPad Prism version 6.07 (GraphPad, San Diego, California) was used for statistical analysis. To determine the standard deviation between the different treated groups, an ANOVA was performed. Unpaired *t*-tests were performed for TLR4 activation measurements using the Hela TLR4 dual reporter cell line. For all data sets based on primary immune cells, paired *t*-tests were carried out. Differences between groups were considered as significant when ^*^p < 0.05, ^**^p < 0.01, or ^***^p < 0.001. The number of experiments performed for each data set is described at the end of each figure legend.

## Results

### Nitration of ATI by Different Nitrating Agents

The degree of ATI nitration varied for the different nitrating agents and methods used. High nitration was observed with TNM and ONOO^−^. Lowest nitration was obtained for O_3_/NO_2_ (Table 1). All three nitration methods were able to modify ATI and induce about 3–13% of covalently linked ATI oligomers (Figures S2A,B).

**Table 1:** Levels of ATI nitration by different nitrating agents.

**Agent**	**Reaction condition**	**Nitration degree (%)^a^**	**Nitrotyrosine [μg/ml]^b^**
TNM	4.55 μL [4%], 3 h	19.5 ± 0.2	0.36 ± 0.03
O_3_/NO_2_	O_3_ [200 ppb], NO_2_ [50 ppb], 4 h	8.6 ± 0.5	7.8E-04 ± 2.4E-04
ONOO^−^	2.85 μl [200mM], 1.8 h	26.2 ± 6,2	0.23 ± 0.12

a *The ND values here were corrected according to the background for pre-nitrated signals of native ATI (n = 2). Blank samples were conducted under the same nitration conditions, and no signal at 280 nm and 357 nm was detected for all the mock samples*.

b *The values are expressed in equivalents of nitrotyrosines [μg] nitrated BSA nBSA per 1 μg of nitrated ATI solution (n = 2). The values were corrected against unmodified ATI solution. Mock samples showed no nitration*.

### Nitration of ATI Enhances TLR4 Stimulatory Activity

**Figure 1:**
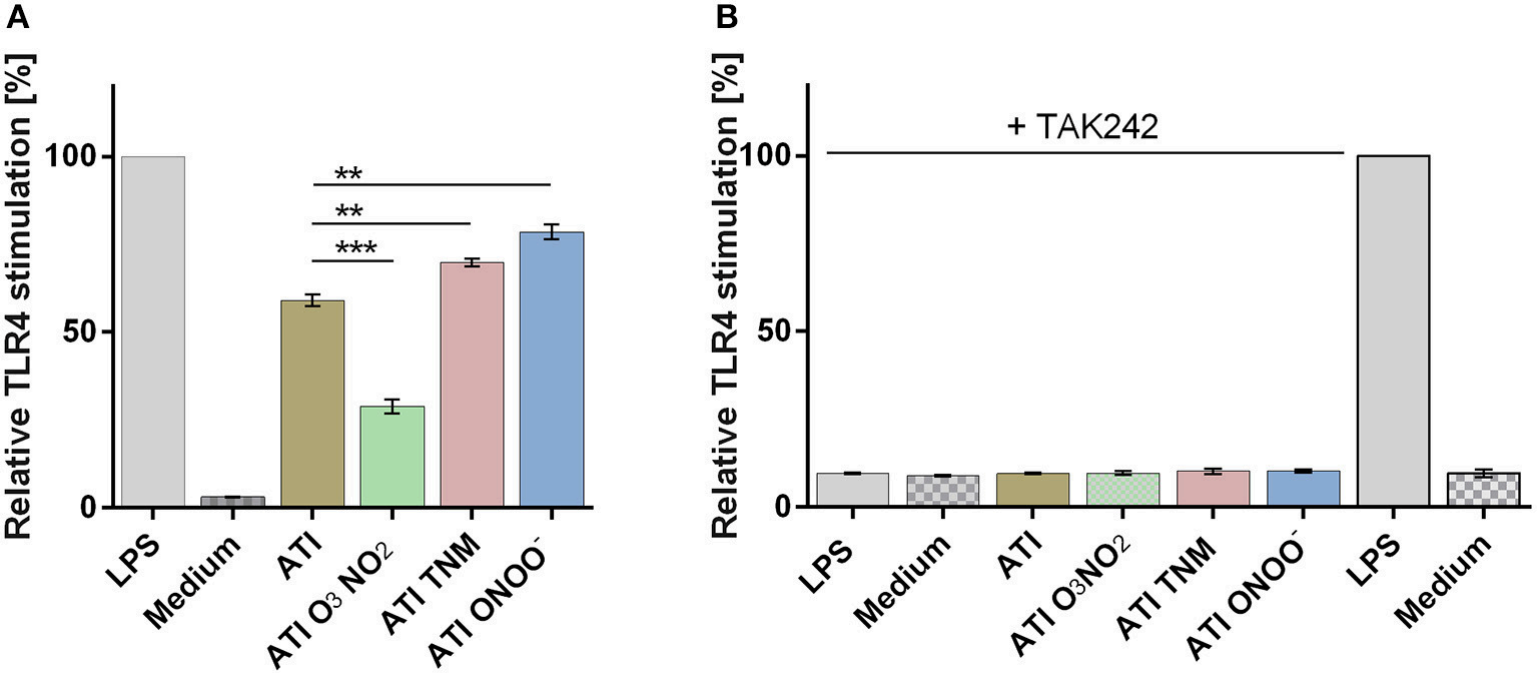
Different nitrated ATI induce distinct TLR4 activation in HeLa TLR4 dual reporter cells. **(A)** HeLa TLR4 dual reporter cells were treated for 7 h with nitrated or unmodified ATI [7.5 μg/mL] or LPS [25 ng/mL] as a positive control. **(B)** Cells were pre-incubated for 2.5 h with TAK242 [0.36 μg/mL] or its solvent DMSO [4.4 μg/mL]. Then the cells were stimulated with nitrated or unmodified ATI [15 μg/mL], or LPS [25 ng/mL] for 7 h. The relative luciferase activity was calculated by dividing the Renilla luciferase (TLR4) signal by the Firefly luciferase (viability) signal. The resulting values were normalized to the value obtained for LPS- treated cells. Shown are the means ± SD of three independent experiments measured in triplicates using two independently nitrated probes. ^***^P < 0.001, ^**^P < 0.01.

Compared to unmodified ATI, TNM, and ONOO^−^, nitrated ATI induced a significantly higher TLR4 activation in HeLa TLR4 dual reporter cells. However, ATI nitrated with O_3_ and NO_2_ exhibited a lower TLR4 activation compared to native ATI (Figure 1A). All mock nitrations showed no TLR4 activation (Figure S3).

Administration of the TLR4 antagonist TAK242 completely diminished TLR4 activation induced by ATI or by the positive control LPS (Figure 1B).

### Nitrated and Unmodified ATI Induce Similar Activation of Macrophages and Immature DC

**Figure 2:**
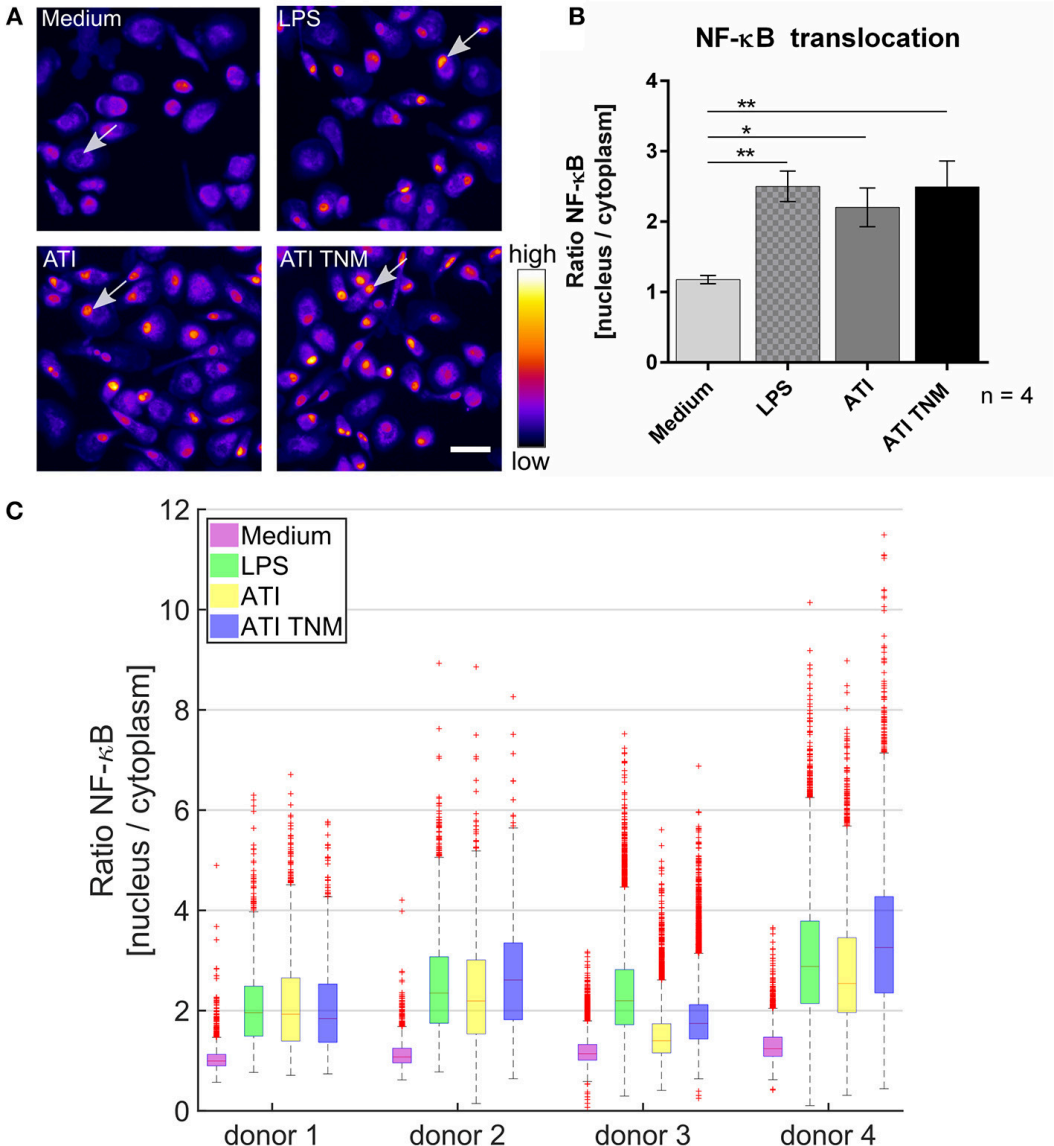
Increased NF-κB p65 translocation into the cell nucleus in ATI and ATI TNM treated primary human macrophages. **(A)** Fluorescence microscopy images of macrophages treated for 2 h with LPS (positive control, 100 ng/mL), ATI or ATI TNM [12.5 μg/mL] and stained for NF-κB. In untreated conditions (medium) NF-κB resides mostly in the cytoplasm, whereas an increased translocation of NF-κB p65 into the nucleus can be observed for LPS, ATI, and ATI TNM treated cells (indicated by arrows). Brightness and contrast were adjusted to the same level for every image within the panel. Scale bar = 50 μm. **(B)** Quantitative evaluation of NF-κB translocation from the cell cytoplasm to the nucleus by Harmony high-content analysis software (Perkin Elmer). Shown are the means ± SEM from four independent experiments/donors, ^*^P < 0.05, ^**^P < 0.01. **(C)** Donor-dependent response to LPS (green), ATI (yellow), and ATI TNM (blue) treatment.

In unstimulated primary human macrophages, NF-κB is mostly located in the cytoplasm. Upon treatment with ATI or TNM nitrated ATI, a significantly enhanced NF-κB translocation from cytoplasm to the cell nucleus was observed using fluorescence microscopy (Figure 2A). Nitrated ATI exhibited only slightly higher NF-κB translocation compared to native ATI, which was also observed in the individual donor dependent responses (Figures 2B,C).

**Figure 3:**
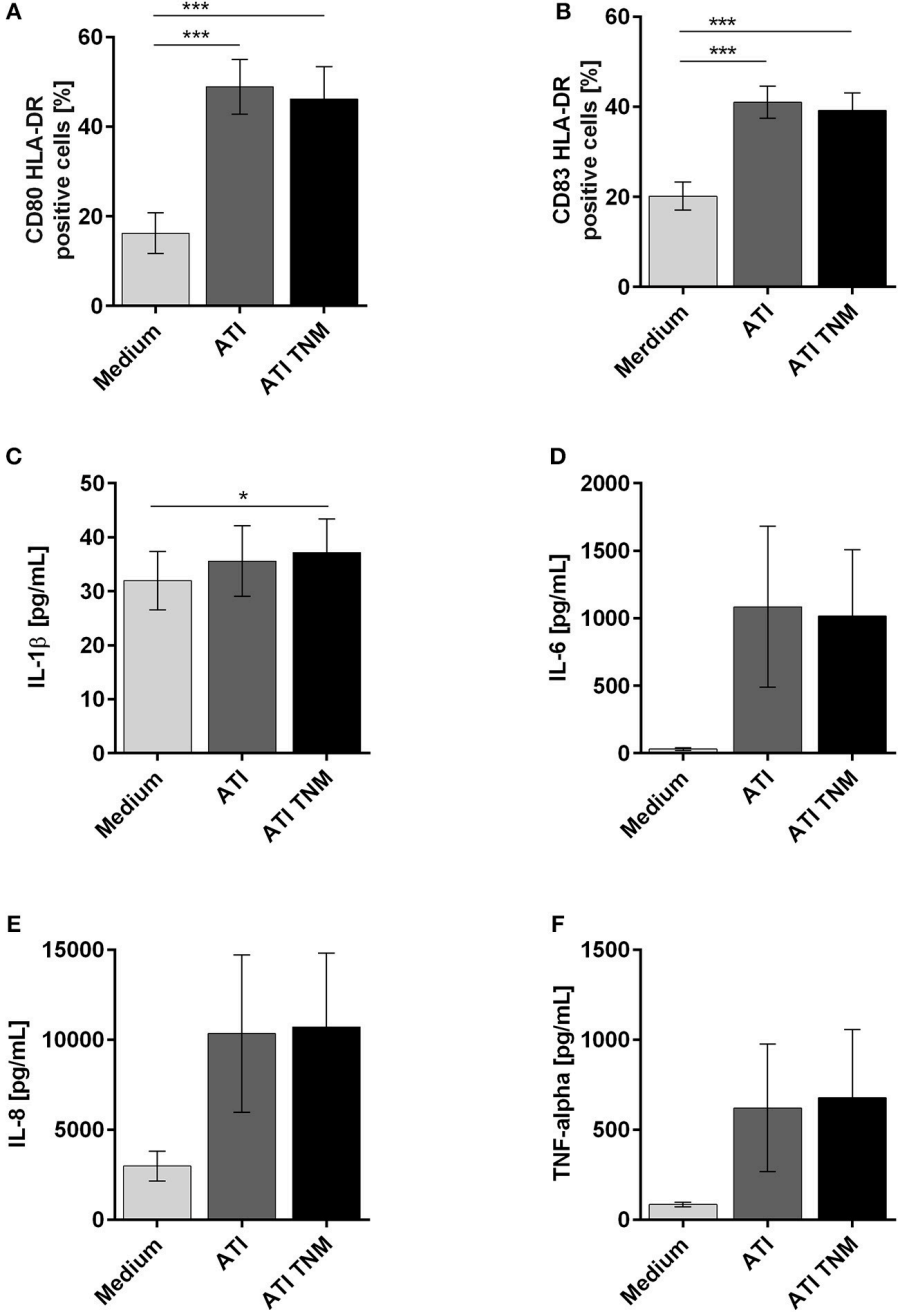
Enhanced expression of maturation markers as well as pro-inflammatory cytokines by human immature DCs upon stimulation with ATI and ATI TNM. Immature DCs were pulsed with ATI or ATI TNM [15 μg/mL] on day 6. Forty-eight hours later cells were stained for expression of the indicated surface markers and analyzed by flow cytometry **(A,B)**. Before, supernatants were taken for determination of IL-1β, IL-6, IL-8, and TNF-alpha by magnetic multiplex assay **(C–F)**. Shown are the means ± SEM from ten independent experiments/donors, ^*^P < 0.05, ^***^P < 0.001.

Immature DC treated with unmodified or TNM nitrated ATI expressed significantly higher amounts of CD80, CD83, and HLA-DR on their surface in comparison to untreated cells (Figures 3A,B). For DC treated with mock nitrated samples, no changes were observed (Figures S4A,B). Furthermore, the release of the pro-inflammatory cytokines IL-1β, IL-6, IL-8, and TNF-alpha by immature DC was similarly elevated upon ATI or ATI TNM treatment compared to untreated cells (Figures 3C–F). No significant changes were observed for MCP-1 release or cells treated with mock nitrations (Figures S4C–G).

### Nitrated ATI Enhance Proliferation and Induce an Enhanced Th1 and Th2 Cytokine Expression Profile in CD4^+^ T Cells Stimulated With Autologous Mature DC

**Figure 4:**
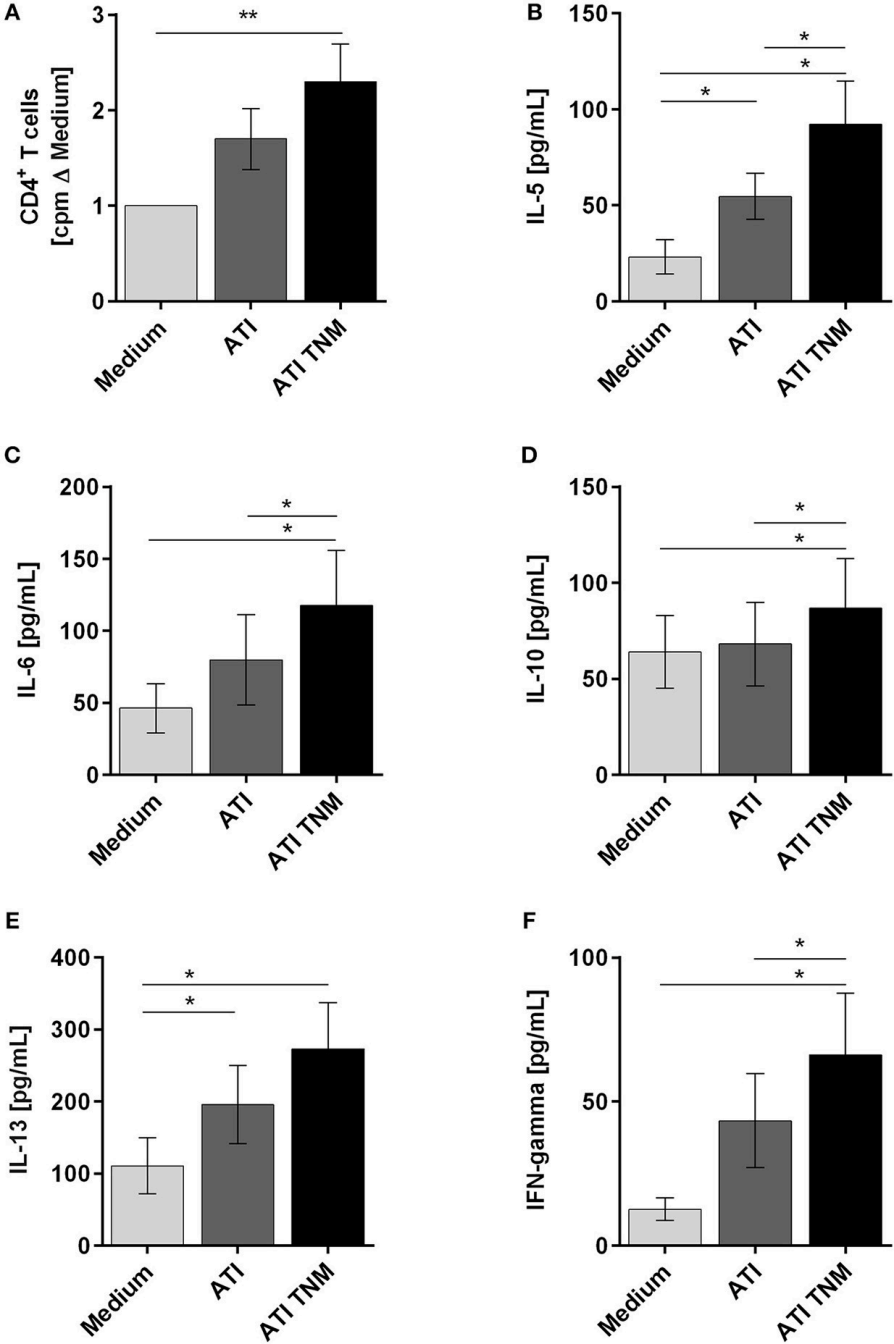
Enhanced T cell proliferation as well as Th1 and Th2 cytokine production of CD4^+^ T cells stimulated with autologous ATI TNM-pulsed mature DC. Immature DC were pulsed with ATI or ATI TNM [15 μg/mL] and matured with pro-inflammatory cytokines as described in Materials and Methods. After 48 h, mature DC were washed and co-cultivated with autologous CD4^+^ T cells for 5 days. **(A)** T cell proliferation was measured by [^3^H]-thymidine incorporation, and proliferation index was calculated related to untreated cells. **(B–F)** Supernatants (50 μL) were collected before thymidine administration to determine the production of IL-5, IL-6, IL-10, IL-13, and IFN-gamma by multiplex assay. Results are presented as means ± SEM from 10 independent experiments/donors. In some cases, cytokine concentrations were below the detection limit so that *n* < 10 (IL-5 *n* = 8, IL-13 *n* = 5, IFN-gamma *n* = 9), ^*^P < 0.05, ^**^P < 0.01.

To analyze the immunogenicity of nitrated vs. unmodified ATI, CD4^+^ T cells were co-cultured with autologous mature DC treated before with ATI or ATI-TNM. Proliferations as well as production of Th1 and Th2 cytokines were analyzed. In general, CD4^+^ T cells from almost all donors showed an enhanced proliferative response and cytokine release after stimulation with ATI-pulsed compared to untreated DC. Only DC treated with nitrated ATI induced a significantly increased T cell proliferation (Figure 4A). Moreover, the release of the Th2 cytokines IL-5, IL-6, IL-10, and IL-13 as well as the Th1 cytokine IFN-gamma was significantly enhanced after stimulation with DC treated with ATI-TNM (Figures 4B–F). Comparing both ATI-treated groups, we found a significant higher release for IL-5, IL-6, IL-10, and IFN-gamma upon the ATI-TNM stimulus (Figures 4B,D,F). There were no significant changes in IL-4 and IL-17A release between all groups (Figures S5A,B). Mock nitration did neither influence cytokine release nor T cell proliferation (Figures S5C–H).

## Discussion

In the present study, we analyzed the impact of nitration on the immunogenicity of ATI, and could show that ATI can be nitrated by exogenous and endogenous nitrating agents. Furthermore, we demonstrated that nitration of ATI lead to enhanced innate and adaptive immune responses compared to unmodified ATI (Graphical Abstract).

**Graphical Abstract:**
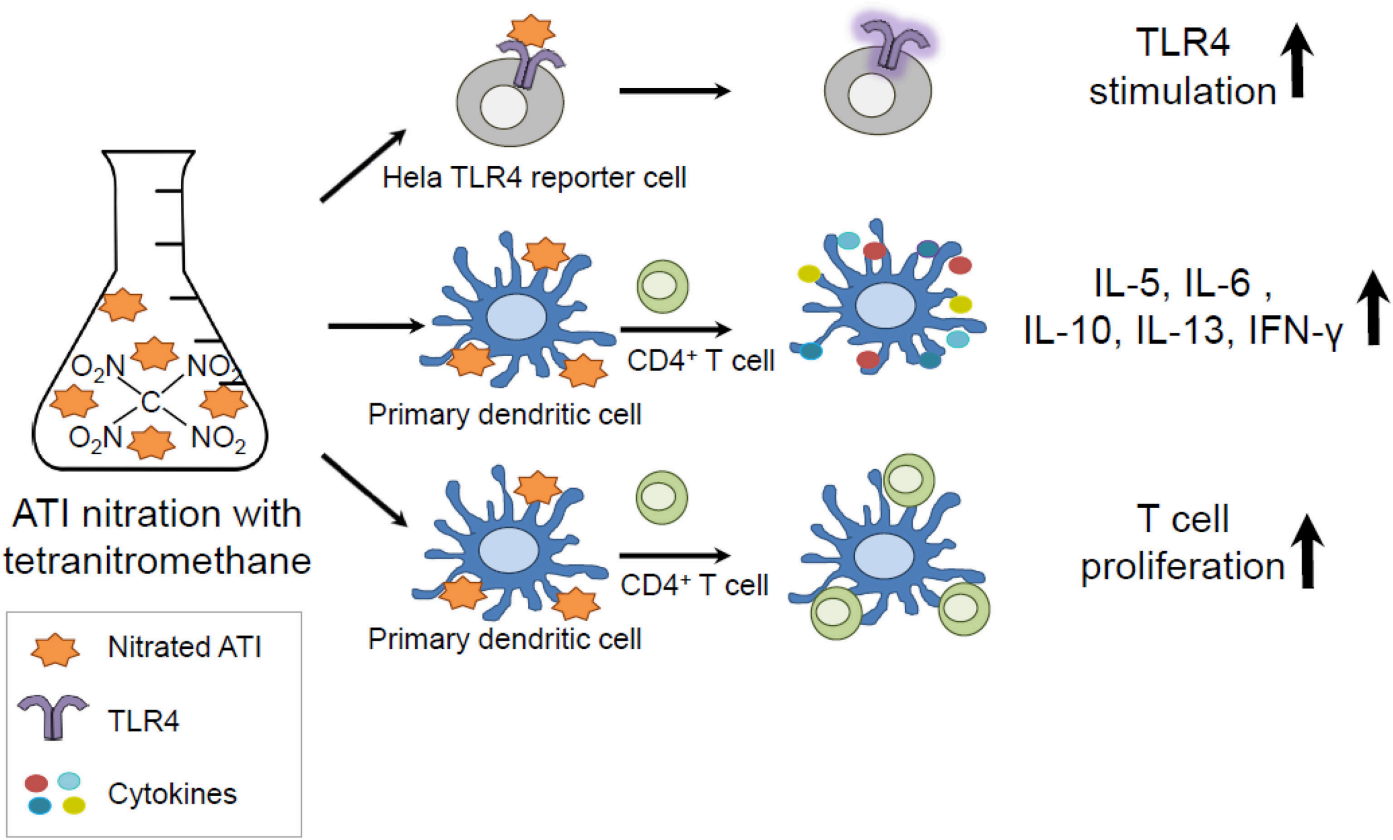
Amylase Trypsin Inhibitors (ATI) can be found in all gluten containing grains and are capable to mediate innate immunity by direct TLR4 stimulation. As protein nitration can occur during inflammatory processes in the body and under environmental conditions, ATI were nitrated and tested *in vitro*. The nitrated ATI induced increased innate and adaptive immune reactions.

By using a novel HeLa TLR4 dual luciferase reporter cell line, we could demonstrate that TNM and ONOO^−^ mediated ATI nitration induced significant enhanced TLR4 stimulation, which seems to correlate with the higher nitration degrees as determined by HPLC-DAD and ELISA. Pre-incubation of the cells with the TLR4 antagonist TAK242 inhibited ATI-induced TLR4 signaling, providing further evidence that ATI stimulate TLR4 directly, as reported in previous studies (21, 22). The complete abolishment of TLR4 activity by TAK242 furthermore allows the conclusion that nitrated ATI also impact the TLR4 agonistic properties directly. As nitration always induces the formation of nitrotyrosine (1, 5, 9, 27–31), it may be hypothesized that nitrotyrosine embedded into the ATI secondary structure itself serves as an amplifier. This hypothesis is supported by our results, showing the lack of enhanced TLR4 stimulation in HeLa TLR4 dual reporter cells upon treatment with unmodified ATI combined with pure nitrotyrosine (Figure S6).

Moreover, nitration of proteins is known to change their structure and function (29). Further investigations would be necessary to clarify to which degree nitration of ATI alters their interaction with TLR4 (22), which may explain the observed enhanced TLR4 agonistic activity.

One essential step in TLR4 signaling is the nuclear translocation of the transcription factor NF-κB, controlling the expression of many genes relevant in innate and adaptive immunity (23, 24, 32). Therefore, we performed several studies on primary immune cells from healthy human donors *in vitro*. Using fluorescence microscopy, we visualized and quantified nuclear translocation of NF-κB in primary human macrophages after the addition of ATI or nitrated ATI. Under the conditions chosen, native and nitrated ATI provoked comparable nuclear translocation of NF-κB.

Previous studies showed that, not only NF-kB, but also the interferon regulatory factor (IRF-3) gets activated upon ATI treatment (21). Therefore, future investigations should also investigate whether nitrated ATI effect the IRF-3 pathway.

Moreover, we found that the NF-κB controlled cytokines IL-1β, IL-6, IL-8, TNF-alpha, and the DC maturation markers CD80 and CD83 (32) were strongly enhanced after treatment of immature DC with ATI and nitrated ATI, which is consistent with previous studies (21, 26). This finding indicates that ATI in general induce the maturation of DC, which is an essential step in the induction of T cell mediated immunity, both via Th1 and Th2 T helper cells, as occurs in autoimmunity and allergy, underpinning the intrinsic immune adjuvant function of (nutritional) ATI (20, 25, 26).

To investigate the modulation of adaptive immunity by nitrated ATI, we analyzed syngeneic DC-T cell co-cultures. Here, only TNM ATI treated DC induced significantly enhanced T cell proliferation. Moreover, treatment of DC with ATI or nitrated ATI caused a significantly higher release of Th2 relevant cytokines IL-5, IL-6, IL-10, and IL-13, and of the Th1 cytokine IFN-gamma. For unmodified ATI, this finding is in line with our prior studies on nutritional and inhalative allergies (25, 26). Remarkably, nitrated ATI significantly enhanced the production of both Th1 and Th2 cytokines compared to unmodified ATI.

Therefore, it can be concluded that nitrated ATI possess an overall enhanced immune stimulatory potential, including Th1 mediated diseases, as was shown by us before for unmodified ATI in murine inflammatory bowel disease (20). These results make it highly probable that nitrated ATI may more strongly promote inhalative wheat allergies (baker's asthma), NCWS, other allergies, and autoimmune diseases.

Similar findings were reported for other nitrated allergens in the context of allergic airway inflammation or anaphylaxis, but the exact underlying molecular mechanisms are still elusive (1–6). Allergen oligomerization as a side product during the nitration process (e.g., cross-linking of protein-bound tyrosine to form a dimer, or the formation of disulfide bridges) was observed for several proteins, and is discussed as one possible mechanism (4, 30, 31, 33). In this respect, it was suggested that one structural aspect of allergens is their capability to form dimers or oligomers, thereby enhancing their allergenicity (34). Ackaert et al. (4) reported two-fold higher stability for nitrated, as compared to unmodified, Bet v 1, making the allergen more resistant to proteolytic degradation, thus permitting enhanced and prolonged presentation on DC, followed by an enhanced T cell response (3, 4). Interestingly, higher molecular weight aggregates instead of oligomers of Bet v 1 (4), and the hypoallergenic Bet v 1d (35) were reported to favor Th1 immunogenicity. Indeed, for all nitration methods used, we found fractions of covalently linked ATI oligomers after the reaction.

Taken together, our results demonstrate that ATI are potent stimulators of innate and adaptive immune responses. In contrast to unmodified ATI, the nitrated ATI exhibit stronger immune stimulatory effects. The observed enhanced immunogenicity of ATI provides a causative chain between stronger TLR4 agonistic effects, leading to enhanced Th1 and Th2 cell activation upon nitration. Future investigations are needed to test how far the degrees of nitration obtained in our study match endogenous ATI nitration under inflammatory conditions, or exogenous nitration, which may occur under certain conditions of wheat growing and processing, e.g., via intensive fertilization, environmental pollution, or nitration during food processing.

## Data Availability Statement

All datasets analyzed for this study are included in the manuscript and the supplementary files.

## Author Contributions

DS, UP, KR-S, IB, and KL designed the experiments. KZ, JN, FL, and IB performed the experiments. KZ, JN, FL, JF-N, DS, UP, IB, and KL analyzed and interpreted the data. CC, JS, FL, KR-S, and IB contributed materials, methods, analysis tools. KZ, IB, and KL wrote the paper. All authors were involved in the editing and proofreading of the manuscript.

### Conflict of Interest Statement

The authors declare that the research was conducted in the absence of any commercial or financial relationships that could be construed as a potential conflict of interest.
