# Supplementary material for: Nitration of Wheat Amylase Trypsin Inhibitors Increases Their Innate and Adaptive Immunostimulatory Potential in vitro
Source: Front Immunol. 2019 Jan 21;9:3174. doi: 10.3389/fimmu.2018.03174 (PMC6357940; doi:10.3389/fimmu.2018.03174)
Supplement: Supplementary file 1 [file Data_Sheet_1.pdf]

Supplementary Material

Nitration of Wheat Amylase Trypsin Inhibitors Increases Their Innate and Adaptive Immunostimulatory Potential *in vitro*

Kira Ziegler[1], Jan Neumann[1,2], Fobang Liu[1], Janine Fröhlich-Nowoisky[1], Christoph Cremer[2,1], Joachim Saloga[3], Kathrin Reinmuth-Selzle[1], Ulrich Pöschl[1], Detlef Schuppan[4], Iris Bellinghausen[3†], and Kurt Lucas[1†*]

[1]*Multiphase Chemistry Department, Max Planck Institute for Chemistry, Mainz, Germany*

[2]*Institute of Molecular Biology, Mainz, Germany*

[3]*Department of Dermatology, University Medical Center of the Johannes Gutenberg University, Mainz, Germany*

[4]*Institute of Translational Immunology, University Medical Center of the Johannes Gutenberg University, Mainz, Germany*

†equally contributing senior authors

*** Correspondence:**

Dr. Kurt Lucas

k.lucas@mpic.de

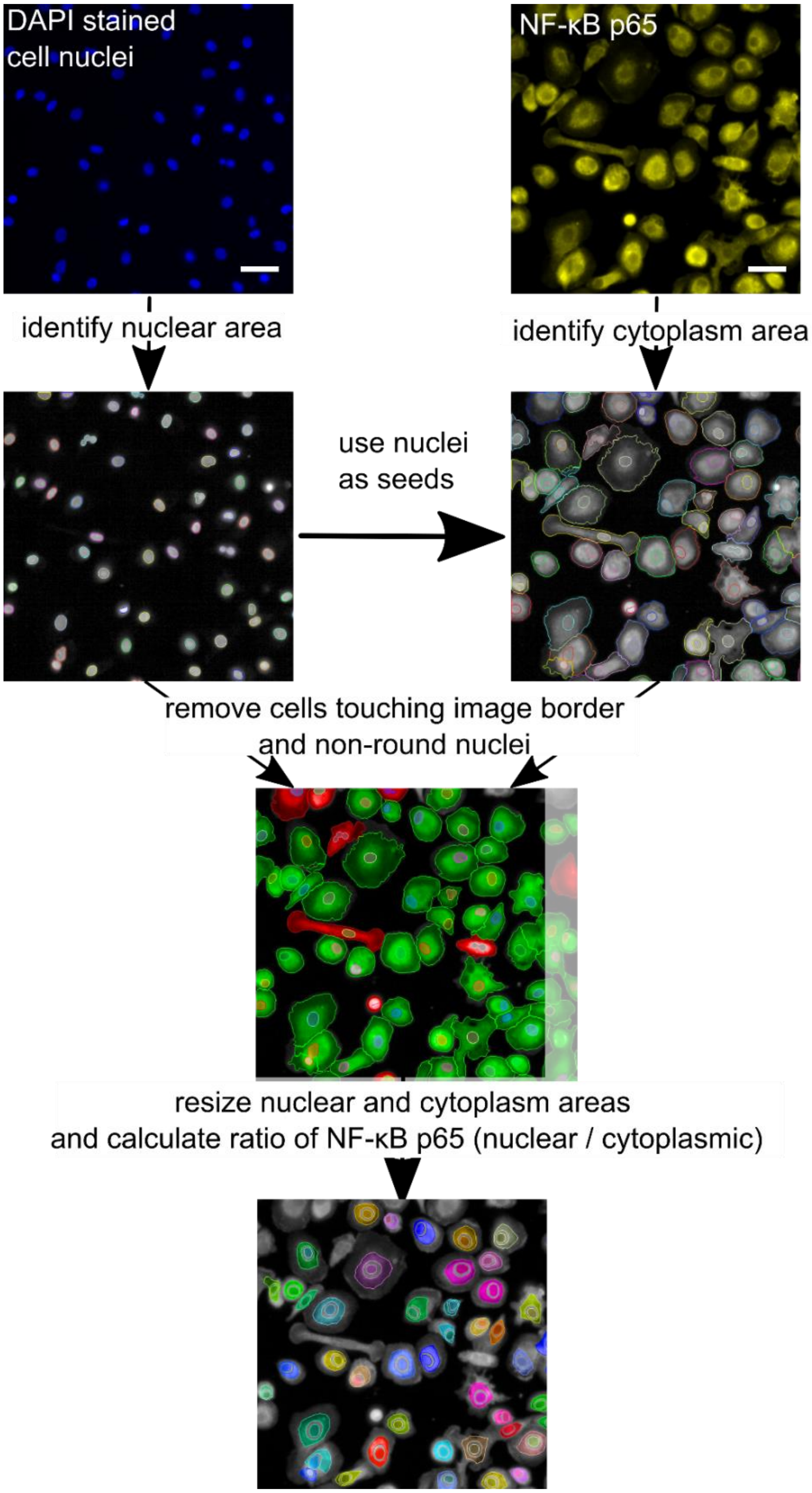

Supplementary Figure 1. Image processing steps to quantify nuclear translocation of NF-κB. DAPI (pseudocolor blue) and NF-κB (pseudocolor yellow) input images were taken at the Opera Phenix High-content screening system (Perkin Elmer, Waltham, Massachusetts, USA). The output image contains the nuclear and cytoplasm masks, which were used to calculate the mean NF-κB p65 intensities within the cytoplasm and the nucleus. Scale bar = 50 µm.

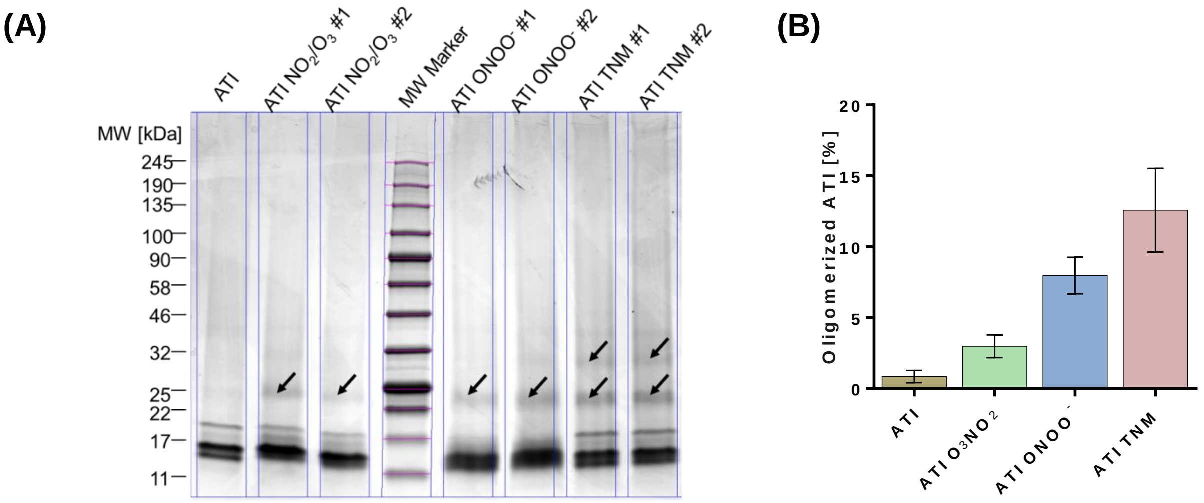

Supplementary Figure 2. SDS PAGE of unmodified ATI and nitrated ATI samples.
5 µg of each ATI sample was analyzed by sodium dodecyl sulfate polyacrylamide gel electrophoresis (SDS PAGE). Molecular weight (MW) is shown in kDa. A: Oligomers can be found for all nitrated ATI samples (indicated with arrows). B: Quantification of oligomers was carried out by Image lab software (Biorad). Results are presented as means ± SEM from n = 2 independent SDS PAGE and nitration experiments.

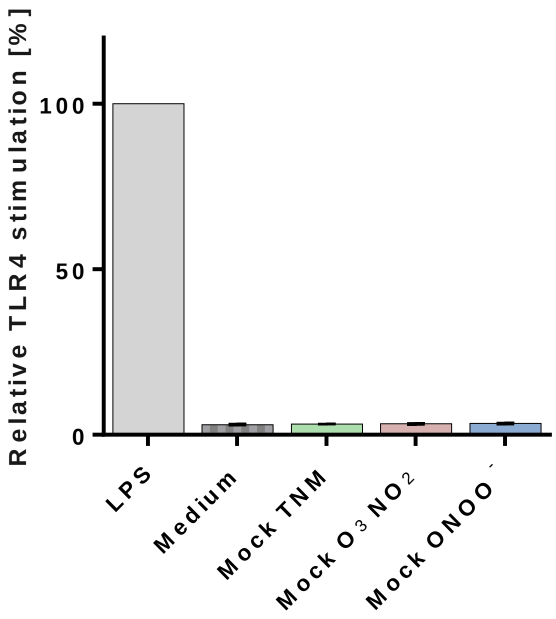

Supplementary Figure 3. TLR4 stimulation by Mock nitration on HeLa TLR4 dual reporter cells.
HeLa TLR4 dual reporter cells were treated for 7 h with equivalent volumes of mock nitrated solution, LPS [25 ng/mL] served as positive control. The relative luciferase activity was calculated by dividing the Renilla luciferase (TLR4) signal to the Firefly luciferase (viability) signal. The resulting values were normalized to the value obtained for LPS treated cells. Shown are the means ± SD of three independent experiments carried out in triplicates, using two independently nitrated probes.

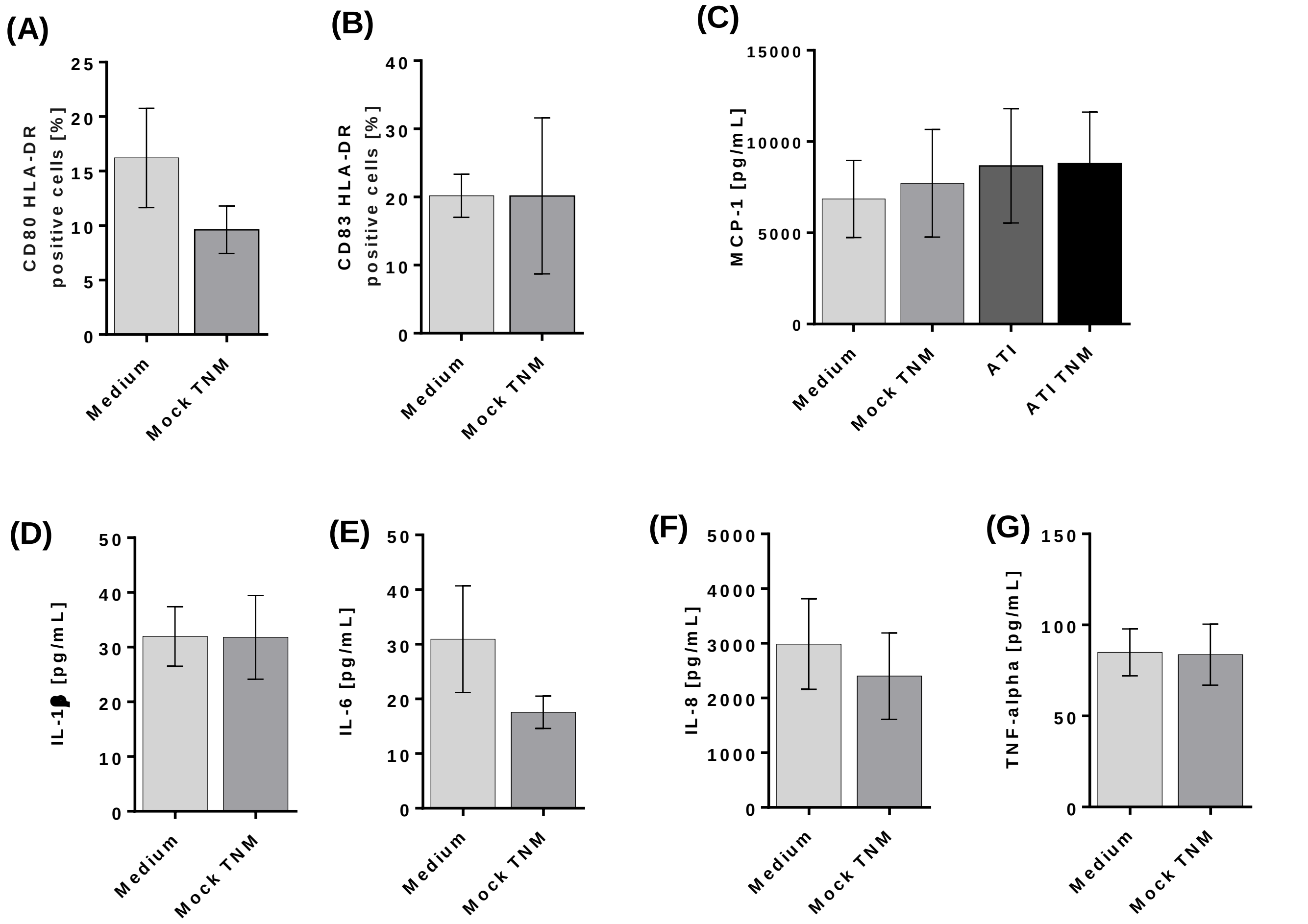

Supplementary Figure 4. Expression of maturation markers as well as pro-inflammatory cytokines by human immature DCs upon stimulation with Mock TNM

Immature DCs were left untreated or pulsed with ATI or ATI TNM [15 µg/mL] or equivalent volumes of mock nitrated solution on day 6. 48 h later the cells were stained for expression of the indicated surface markers and analyzed by flow cytometry (A+B). Before, supernatants were taken for determination of MCP-1, IL-1β, IL-6, IL-8 and TNF-alpha by magnetic multiplex assay (C-G). Shown are the means ± SEM from $n \geq 6$ independent experiments/donors, *P<0.05, ***P<0.001.

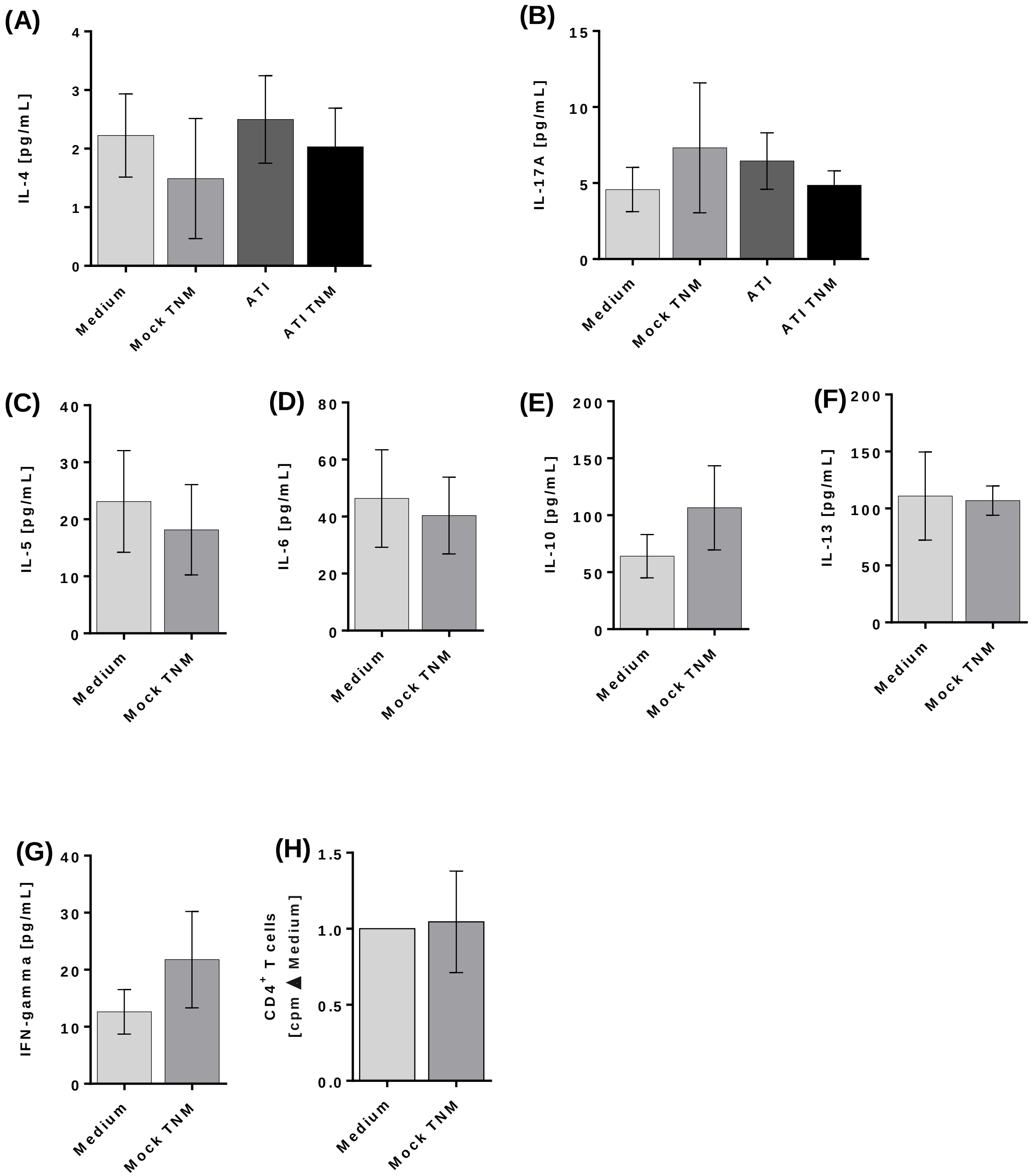

Supplementary Figure 5. T cell proliferation as well as Th1 and Th2 cytokine production of CD4$^+$ T cells stimulated with autologous Mock TNM pulsed mature DC.
Immature DC were left untreated or pulsed with ATI or ATI TNM [15 µg/mL] or equivalent volumes of mock nitrated sample and matured with pro-inflammatory cytokines as described in Materials and Methods. After 48 h, mature DC were washed and co-cultivated with autologous CD4$^+$ T cells for 5 days. A-G: Supernatants (50 µL) were collected before thymidine administration to determine the production of IL-4, IL-17A, IL-5, IL-6, IL-10, IL-13 and IFN-gamma by multiplex assay. H: T cell

proliferation was measured by [^{3}H]-thymidine incorporation and proliferation index was calculated related to untreated cells. Results are presented as means ± SEM from $n \geq 6$ independent experiments/donors.

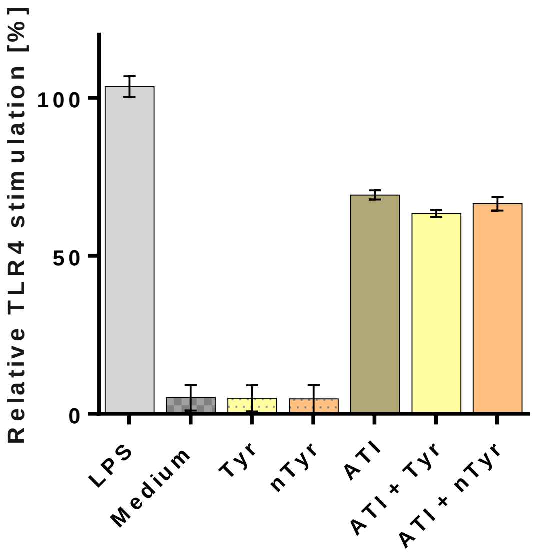

Supplementary Figure 6. TLR4 stimulation by ATI combined with pure nitrotyrosine.

HeLa TLR4 dual reporter cells were treated for 7 h with ATI [7.5 µg/ml] with or without addition of tyrosine or nitrotyrosine [30 µM], LPS [25 ng/mL]. The relative luciferase activity was calculated by dividing the Renilla luciferase (TLR4) signal to the Firefly luciferase (viability) signal. The resulting values were normalized to the value obtained for LPS treated cells. Shown are the means ± SD two independent experiments carried out in duplicates.
